# Supplementary material for: A recurrent neural network model of prefrontal brain activity during a working memory task
Source: PLoS Comput Biol. 2023 Oct 18;19(10):e1011555. doi: 10.1371/journal.pcbi.1011555 (PMC10615291; doi:10.1371/journal.pcbi.1011555)
Supplement: S1 Note — (DOCX) [file pcbi.1011555.s001.docx]

**S1 Note. Complementary analyses for networks from Experiment 1.**

**Decoding of the pre-cue, uncued and cued items**

To verify the results of the CDI analysis reported in **Fig 3H** whilst accounting for the noise in the colour representations, we performed an analogous decoding analysis. We trained binary LDA classifiers to discriminate between colour pairs in two-fold cross-validation. To quantify colour decoding in the pre-cue delay, classifiers were trained to discriminate between colours from a single location using the data from the endpoint of the pre-cue delay and tested on withheld trials from the same condition. Similarly, to quantify the cued item decoding accuracy, classifiers were trained to discriminate between cued colours on a subset of trials where e.g. location 1 was cued, and tested on the withheld trials from the same condition. We used the same approach to quantify the cross-validated decoding accuracy for the uncued item. We found that the pre-cue colour test decoding accuracy was near ceiling (M = 99.4% across all colour pairs, locations, cross-validation folds and models). We performed the same contrasts as described in the main text for the CDI analysis. Cued item test decoding accuracy was not significantly higher than the pre-cue baseline (M = 99.4%, Wilcoxon signed-rank test W(29) = 26, p = .917, matched rank biserial correlation r = .205), but it was significantly higher than the uncued item test decoding accuracy (M = 98.2%; Wilcoxon signed-rank test *W*(29) = 465, *p* < .001, matched rank biserial correlation r = -1). To test our hypothesis of no difference between the uncued and pre-cue item CDI, we performed a Bayesian paired samples t-test. We repeated the same analysis with the test decoding scores, which returned a BF_10_ of 922037.11, providing strong evidence for the alternative hypothesis of a significant difference between the pre-cue and uncued colour decoding, Overall, this pattern of results contrasts with those reported for the CDI metric, where we found evidence for a selective increase in the cued item CDI relative to the pre-cue baseline, and no change in the uncued item CDI. In contrast, the decoding results described here suggest no statistically significant increase in the decoding of the cued item relative to the pre-cue baseline, and a drop in the uncued item decoding. It is however important to note that the colour decoding accuracy in the pre-cue delay was already very close to ceiling, thus limiting the scope for an increase in the decoding of the cued item in the post-cue delay.

**Cued plane transformations quantified with plane angles**

As reported in the main text, our results suggested that only one of the memory representations was rotated after the cue (**Fig 2H**). To further investigate this finding, we repeated the cross-validated AI analysis described in the main text, this time calculating the between-plane angles $\theta$ and phase-alignment angles $\psi$. In short, we used half the data to identify the *unrotated* and *rotated* locations (based on the absolute cos($\theta)$ value between the pre-cue and post-cue Cued planes) and the other half to confirm whether there were any significant differences in $\theta$ and $\psi$ between them. Focusing first on $\theta,$ we rectified the rotated and unrotated angles (**S1D Fig)** and calculated the difference between them. We found evidence for significant clustering in this difference (Rayleigh test: *z*(29) = 16.59, *p* < .001) with a mean of 24.93°, indicating that $\theta$ for the rotated plane was on average higher than the one for the unrotated plane. This is in agreement with the AI results from **Fig 2H**. With respect to the phase-alignment between the pre-cue and post-cue planes, we found both the unrotated and rotated $\psi$ to be significantly clustered (Rayleigh test: *z*(27) = ﻿27.98, *p* < .001 and *z*(23) = 23.98, *p* < .001, respectively), with the angular means of 0.46° and 0.18°, respectively.

**Single readout hypothesis**

To formally check if the same linear readout could be used to discriminate between colours irrespective of the cued location, we trained binary LDA classifiers on hidden activity data from the last timepoint of the trial (in 2-fold cross-validation) and tested them on (1) the withheld trials on which the same location was cued (to check the decoding test accuracy) and (2) all the trials on which the other location was cued (to see if the classifier would cross-generalise to the other plane). Mean test decoding accuracy (1) was 99.35% (averaged across individual classifiers, cross-validation folds, datasets (defined by the cued location) and models). It was significantly higher than chance (50%) across all models, as evidenced by the results of a one-tailed Wilcoxon signed-rank test: W(29) = 465, p < .001 (Cohen’s d = 63.18). Mean cross-generalisation decoding accuracy (2) was 83.08% and was also significantly higher than expected by chance (one-sample one-tailed t-test: t(29) = 19.59, p < .001; Cohen’s d = 3.58).
