# Supplementary material for: A recurrent neural network model of prefrontal brain activity during a working memory task
Source: PLoS Comput Biol. 2023 Oct 18;19(10):e1011555. doi: 10.1371/journal.pcbi.1011555 (PMC10615291; doi:10.1371/journal.pcbi.1011555)
Supplement: S2 Note — (DOCX) [file pcbi.1011555.s002.docx]

**S2 Note. Training speed comparison for networks trained under various post-cue maintenance pressure conditions.**

We examined the training time of networks (quantified as the number of training epochs completed until loss threshold was reached, NETC) from experiment 2 and found it to differ depending on the length of the post-cue delay (one-way ANOVA with factor post-cue delay length (8 levels) with log-transformed NETC as the dependent variable, F(7,232) = 50.74, p <.001, η² = 0.61). A linear contrast confirmed that the training time declined alongside the post-cue delay length (t(232) = -18.66, p < .001). These results suggest that networks using the orthogonal (short post-cue delay) solution need to complete the most training iterations to converge, as compared to the networks using parallel plane solutions (trained with long post-cue delays).
