# Supplementary material for: A recurrent neural network model of prefrontal brain activity during a working memory task
Source: PLoS Comput Biol. 2023 Oct 18;19(10):e1011555. doi: 10.1371/journal.pcbi.1011555 (PMC10615291; doi:10.1371/journal.pcbi.1011555)
Supplement: S3 Note — (DOCX) [file pcbi.1011555.s003.docx]

**S3 Note. *Rotated* / *unrotated* Cued plane AI analysis for Experiment 3**

We repeated the *rotated* / *unrotated* Cued plane AI analysis (shown for Experiment 1 in **Fig 2H**) for the variable delays conditions and found the results to be in agreement with those reported for Experiment 1 (fixed delay length networks). More specifically, the mean AI for the rotated plane was significantly lower (2D: AI = 0.24, 3D AI = 0.27) than for the unrotated plane (2D: mean AI = 0;.49, paired sample t-test: t(29) = -8.03, p < .001, Cohen’s d = -1.47, 3D: mean AI = 0.50, paired sample t-test: t(29) = -7.78, p < .001, Cohen’s d = -1.42). The same analysis with plane angles $\theta$ revealed that the mean for the rotated plane is ~54° whereas that for the unrotated plane is close to zero. This difference was statistically significant across networks (Rayleigh test, z(29) = 22.48, p < .001). With respect to the phase-alignment between the pre-cue and post-cue planes, we found both the unrotated and rotated $\psi$ to be significantly clustered (Rayleigh test: z(25) = 26.00, p < .001 and z(24) = 25.00, p < .001, respectively), with the angular means of 0.03° and -0.02°, respectively (see also **S3E Fig**).
