# Supplementary material for: A recurrent neural network model of prefrontal brain activity during a working memory task
Source: PLoS Comput Biol. 2023 Oct 18;19(10):e1011555. doi: 10.1371/journal.pcbi.1011555 (PMC10615291; doi:10.1371/journal.pcbi.1011555)
Supplement: S4 Note — (DOCX) [file pcbi.1011555.s004.docx]

**S5 Note. Results of the ANOVA analyses of mixture model parameters fit to choice data from Experiment 4.**

As noted in the main text, we compared the mixture model parameters fit to the choice data generated by models from Experiment 4 with 2x2 mixed model ANOVAs (or non-parametric equivalents in the case of the probability parameters). The full results of this analysis are described below (data shown in **Fig 7C).**

The memory precision parameter (*K*) showed a significant main effect of trial type (*F*(1,58) = 144.91, *p* < .001; valid = 26.9, invalid = 22.78) and a significant interaction with condition (*F*(1,58) = 144.76, *p* < .001), but no main effect of condition (*F*(1,58) = 0.86, *p* = .358). Post-hoc tests with Holm-correction revealed that recall precision was significantly higher on valid than invalid trials in the 75% validity condition (*t*(29) = 17.02, *p* < .001), whilst there was no difference in the 50% validity condition (*t*(29) = .004, *p* = .997). Furthermore, the precision parameter on invalid trials was significantly lower in the former compared to latter condition (*t*(29) = -4.22, *p* < .001), with an opposite trend on valid trials, which did not however reach statistical significance (*t*(29) = 2.40, *p* = .057).

An analogous analysis concerning the probability of recalling the target item (*pT*) revealed that it was significantly higher on valid than invalid trials (Wilcoxon signed-rank test, *W(59)* = 1337, p = .002; valid = .996, invalid = .994) as well as in the 75%, compared to 50% validity condition (Mann-Whitney U-test: *W*(29) = 286, *p* = .015; *pT* = .997 and *pT* = .992, respectively). There was also a significant interaction between the two factors (mean difference between valid and invalid trials – 75% validity: .003, 50% validity: 6.02 x 10^-6^, Mann-Whitney U-test: *W*(29) = 208, *p* < .001). Holm-corrected post-hoc tests revealed that *pT* was significantly higher on valid trials in the probabilistic than in the neutral condition (Mann-Whitney U-test: W(58) = 199, p < .001), with no significant difference between the conditions on invalid trials (Mann-Whitney U-test: W(58) = 336, p = .187). In the 50% validity condition, there was no significant difference between the two trial types (Wilcoxon signed-rank test: W(29) = 224, p = .871). In contrast, in the 75% validity condition, *pT* was significantly higher on valid than invalid trials (Wilcoxon signed-rank test: W(29) = 430, p < .001).

Probability of random guesses (*pU*) was significantly lower on valid than invalid trials (Wilcoxon signed-rank test, *W(59)* = 571, p = .011) and in the 75%, compared to the 50% validity condition (Mann-Whitney U-test: *W*(29) = 618, *p* = .013). The interaction between the trial type and condition was also significant, with a more pronounced difference between valid and invalid trials in the 75% validity condition (Mann-Whitney U-test: *W*(29) = 637, *p* = .005; *pU* = -.002 and *pU* = -7.810 x 10^-5^ for 75% and 50% validity, respectively). Holm-corrected post-hoc tests revealed a significant between-condition difference on valid (Mann-Whitney U-test: *W*(58) = 683, *p* = .002, *pU* = 0.007 and 0.001 for 50 and 75% validity, respectively) but not invalid trials (Mann-Whitney U-test: *W*(58) = 588, *p* = .084). In the 75% validity condition, *pU* was significantly lower on valid than invalid trials (Wilcoxon signed-rank test, *W(29)* = 75, *p* = .004; *pU* = 0.001 and 0.003, respectively), whilst there was no such difference in the 50% validity condition (Wilcoxon signed-rank test, *W(29)* = 234, *p* = .984).

Lastly, the analogous analysis regarding the probability of recalling the non-target item (*pNT*) yielded a significant main effect of trial type (Wilcoxon signed-rank test, *W(59)* = 577, *p* = .013, valid = 4.68 x 10^-4^, invalid = 8.89 x 10^-4^) but no main effect of the validity condition (Mann-Whitney U-test: *W*(29) = 447, *p* = .971). The interaction between the two factors was significant (mean difference in *pNT* between valid and invalid trials – 75% validity: -9.14 x 10^-4^, 50% validity: 7.0 x 10^-5^, Mann-Whitney U-test: *W*(29) = 628, *p* = .008). Holm-corrected post-hoc tests revealed a significant between-condition difference on valid (Mann-Whitney U-test: *W*(58) = 659, *p* = .006, *pNT* = 6.65 x 10^-4^ and 0.001 for 50 and 75% validity, respectively), but not invalid trials (Mann-Whitney U-test: *W*(58) = 410, *p* = .562). In the 75% validity condition, *pNT* was significantly lower on valid than invalid trials (Wilcoxon signed-rank test, *W(29)* = 75, *p* = .002; *pNT* = 1.99 x 10^-4^ and .001, respectively), whilst there was no such difference in the 50% validity condition (Wilcoxon signed-rank test, *W(29)* = 225, *p* = .887).

In summary, we found significant interaction effects between the validity condition and trial type for all mixture model parameters examined. In the 50% validity condition, there were no significant trial-type differences in the fitted parameters. In contrast, in the 75% validity condition, both the precision (*K*) and probability of recall (*pT*) of target items were significantly higher on valid than invalid trials, whilst the probability of recalling the non-target item (*pNT*) and making random guesses (*pU*) followed the opposite pattern. It is however worth noting that all the estimated probability quantities were close to ceiling (or floor, in the case of *pNT* and *pU*).
