# Supplementary material for: A recurrent neural network model of prefrontal brain activity during a working memory task
Source: PLoS Comput Biol. 2023 Oct 18;19(10):e1011555. doi: 10.1371/journal.pcbi.1011555 (PMC10615291; doi:10.1371/journal.pcbi.1011555)
Supplement: S1 Fig — A. Training dynamics of the networks from Experiment 1. Training loss values across all epochs, colours correspond to individual networks. B. Upper: Raw (black) and smoothed (green) training loss plotted across epochs. Lower: The derivative of the smoothed loss with respect to time. Red dot in both panels corresponds to the local minimum chosen as the ‘mid-training plateau’ timepoint (refer to the Methods section in the main text for details). Data from an example model. C. Results of a decoding analysis complementary to the CDI analysis reported in Fig 3H. Decoding accuracy (expressed as units of standard normal distribution, snd) for the pre-cue subspaces (averaged across both locations, black circles) and cued and uncued subspaces in the post-cue delay (blue triangles and green crosses, respectively). Bar height corresponds to the mean across all models. Significance and Bayes factor values correspond to the results of contrasts reported in the section below. D. Between-plane angles θ (rectified, panels on the left) and phase-alignment angles ψ (panels on the right) for the unrotated (triangles) and rotated (squares) planes. Individual models and population mean shown as transparent and opaque markers, respectively. (DOCX) [file pcbi.1011555.s005.docx]

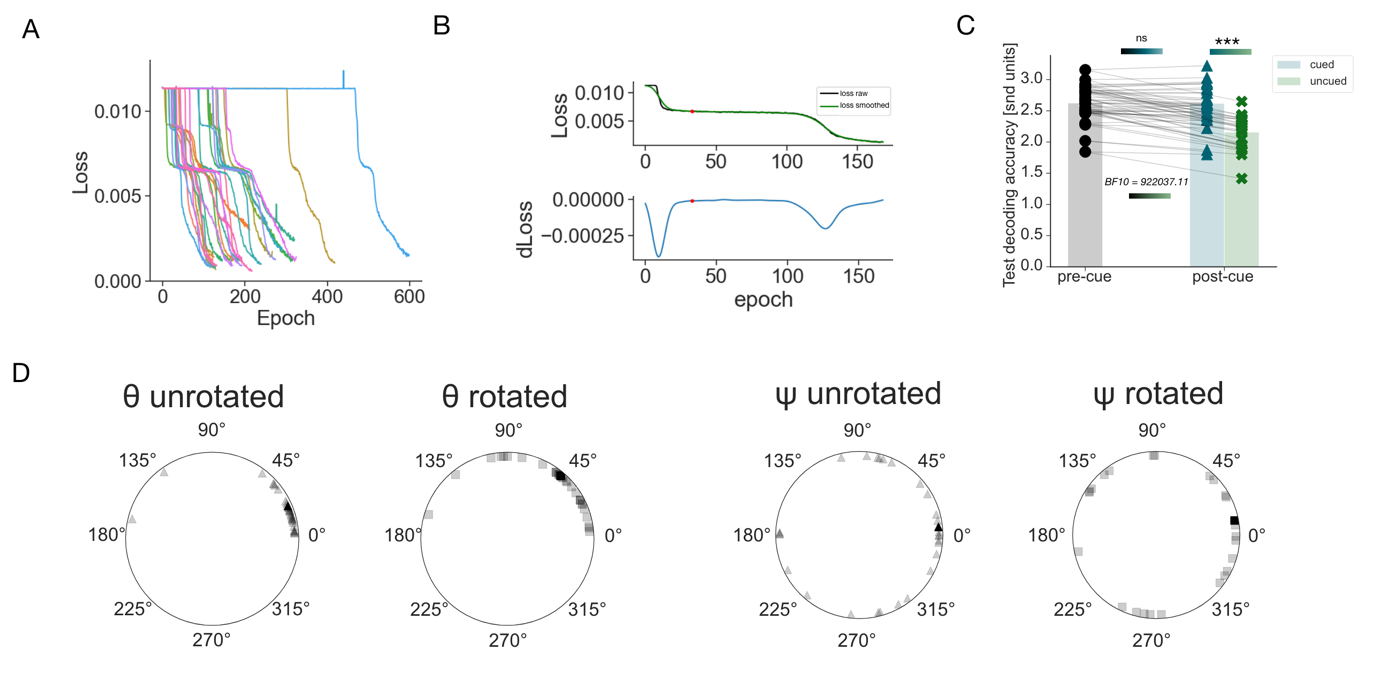


**S1 Fig. Training dynamics and neural geometry of networks from Experiment 1. A.** Training dynamics of the networks from Experiment 1. Training loss values across all epochs, colours correspond to individual networks. **B.** *Upper:* Raw (black) and smoothed (green) training loss plotted across epochs. *Lower:* The derivative of the smoothed loss with respect to time. Red dot in both panels corresponds to the local minimum chosen as the ‘mid-training plateau’ timepoint (refer to the *Methods* section in the main text for details). Data from an example model. **C.** Results of a decoding analysis complementary to the CDI analysis reported in **Fig 3H**. Decoding accuracy (expressed as units of standard normal distribution, *snd*) for the pre-cue subspaces (averaged across both locations, black circles) and cued and uncued subspaces in the post-cue delay (blue triangles and green crosses, respectively). Bar height corresponds to the mean across all models. Significance and Bayes factor values correspond to the results of contrasts reported in the section below. **D.** Between-plane angles $\theta$ (rectified, panels on the left) and phase-alignment angles $\psi$ (panels on the right) for the unrotated (triangles) and rotated (squares) planes. Individual models and population mean shown as transparent and opaque markers, respectively.
