# Supplementary material for: A recurrent neural network model of prefrontal brain activity during a working memory task
Source: PLoS Comput Biol. 2023 Oct 18;19(10):e1011555. doi: 10.1371/journal.pcbi.1011555 (PMC10615291; doi:10.1371/journal.pcbi.1011555)
Supplement: S2 Fig — A. Time to convergence (number of training epochs, y-axis) for models trained with different post-cue delay lengths (x-axis). Dots correspond to the means across models, error bars to SEM. (DOCX) [file pcbi.1011555.s006.docx]

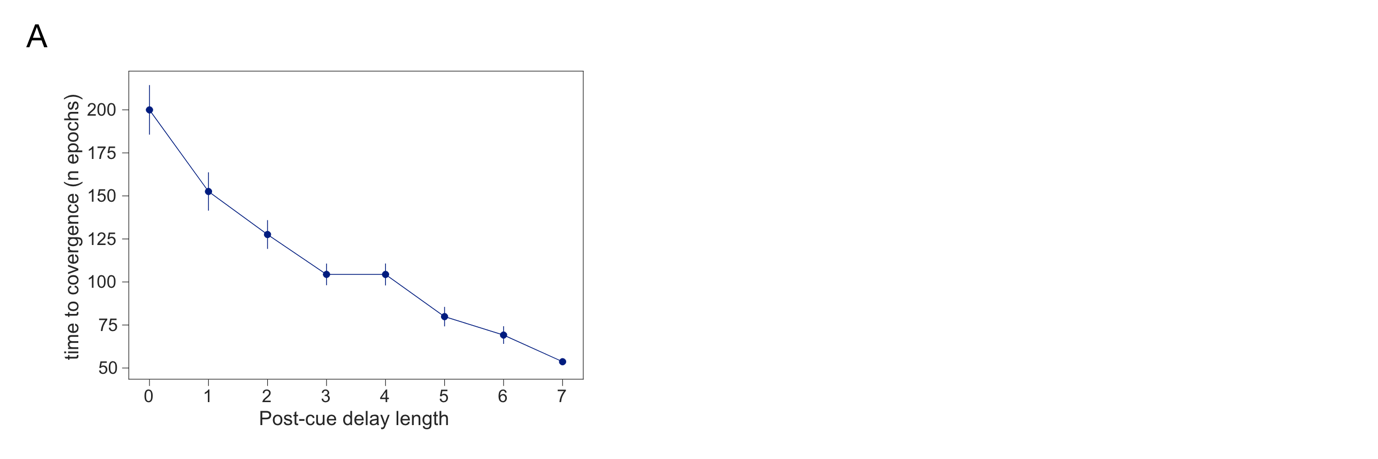


**S2 Fig. Training speed comparison for networks trained under various post-cue maintenance pressure conditions. A.** Time to convergence (number of training epochs, y-axis) for models trained with different post-cue delay lengths (x-axis). Dots correspond to the means across models, error bars to SEM.
