# Supplementary material for: A recurrent neural network model of prefrontal brain activity during a working memory task
Source: PLoS Comput Biol. 2023 Oct 18;19(10):e1011555. doi: 10.1371/journal.pcbi.1011555 (PMC10615291; doi:10.1371/journal.pcbi.1011555)
Supplement: S3 Fig — A. Angular error density plots for the 3 different test conditions: datasets with the delay interval length used during training (trained), a previously not experienced delay length that fell within the training range (in-range), and a novel delay outside of the training range (out-of-range). First and second rows correspond to the data from the networks trained with a fixed delay and variable delay intervals, respectively. Datapoints correspond to M±SEM across all models, shown with the best von Mises fit (solid line). B.-C. Angles θ and ψ between the two Uncued and Cued/Uncued planes (averaged across the two retrocue locations) in the post-cue delay interval, respectively. Individual models shown in transparent, grand averages in opaque colours. D. AI for the unrotated (light grey) and rotated (dark grey) subspaces. Significance values correspond to the results of a paired t-test. E. Analogous plane angles θ and phase alignment angles ψ for the unrotated (triangles) and rotated (squares) planes. Values for individual models and grand averages shown as transparent and opaque markers, respectively. Note theta angles have been rectified. (DOCX) [file pcbi.1011555.s007.docx]

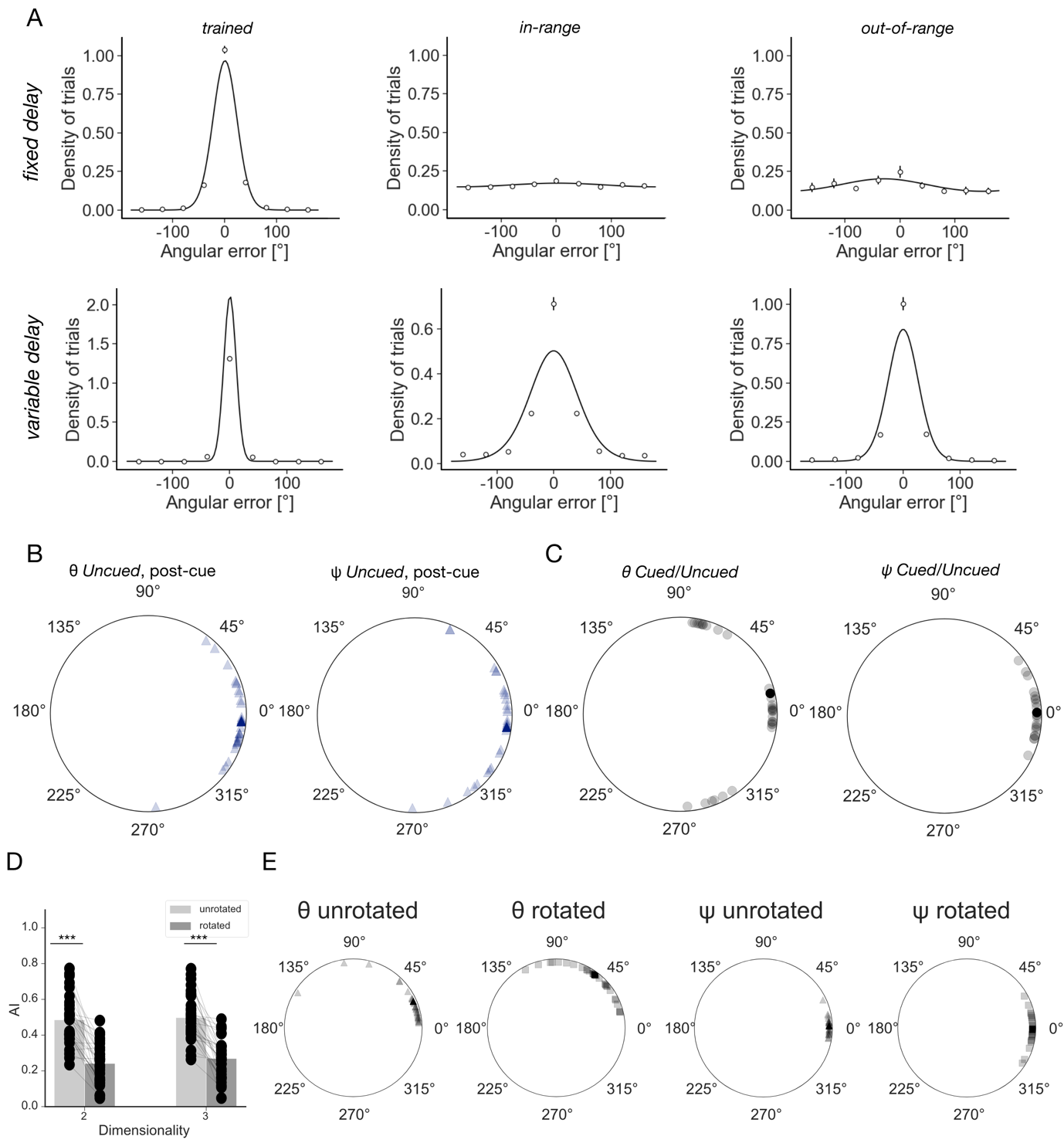


**S3 Fig. Networks trained with variable delay interval lengths show improved temporal generalisation on the task but similar representational geometry as those trained with fixed delay lengths. A.** Angular error density plots for the 3 different test conditions: datasets with the delay interval length used during training (*trained*), a previously not experienced delay length that fell within the training range (*in-range*), and a novel delay outside of the training range (*out-of-range*). First and second rows correspond to the data from the networks trained with a fixed delay and variable delay intervals, respectively. Datapoints correspond to *M*±*SEM* across all models, shown with the best von Mises fit (solid line). **B.-C.** Angles $\theta$ and $\psi$ between the two *Uncued* and *Cued*/*Uncued* planes (averaged across the two retrocue locations) in the post-cue delay interval, respectively. Individual models shown in transparent, grand averages in opaque colours. **D.** AI for the *unrotated* (light grey) and *rotated* (dark grey) subspaces. Significance values correspond to the results of a paired t-test. **E.** Analogous plane angles $\theta$ and phase alignment angles $\psi$ for the *unrotated* (triangles) and *rotated* (squares) planes. Values for individual models and grand averages shown as transparent and opaque markers, respectively. Note theta angles have been rectified.
