# Supplementary material for: A recurrent neural network model of prefrontal brain activity during a working memory task
Source: PLoS Comput Biol. 2023 Oct 18;19(10):e1011555. doi: 10.1371/journal.pcbi.1011555 (PMC10615291; doi:10.1371/journal.pcbi.1011555)
Supplement: S4 Fig — Results of the regression analyses predicting the number of training epochs (after a box-cox transformation) from AI Cued, AI Cued/Uncued and AI Uncued (columns, left to right) in Experiment 4. Rows correspond to the three retrocue validity conditions examined (100, 75 and 50%, top to bottom). Data shown in grey dots, regression lines of best fit in red, 95% confidence interval for the slope in navy dashed lines. (DOCX) [file pcbi.1011555.s008.docx]

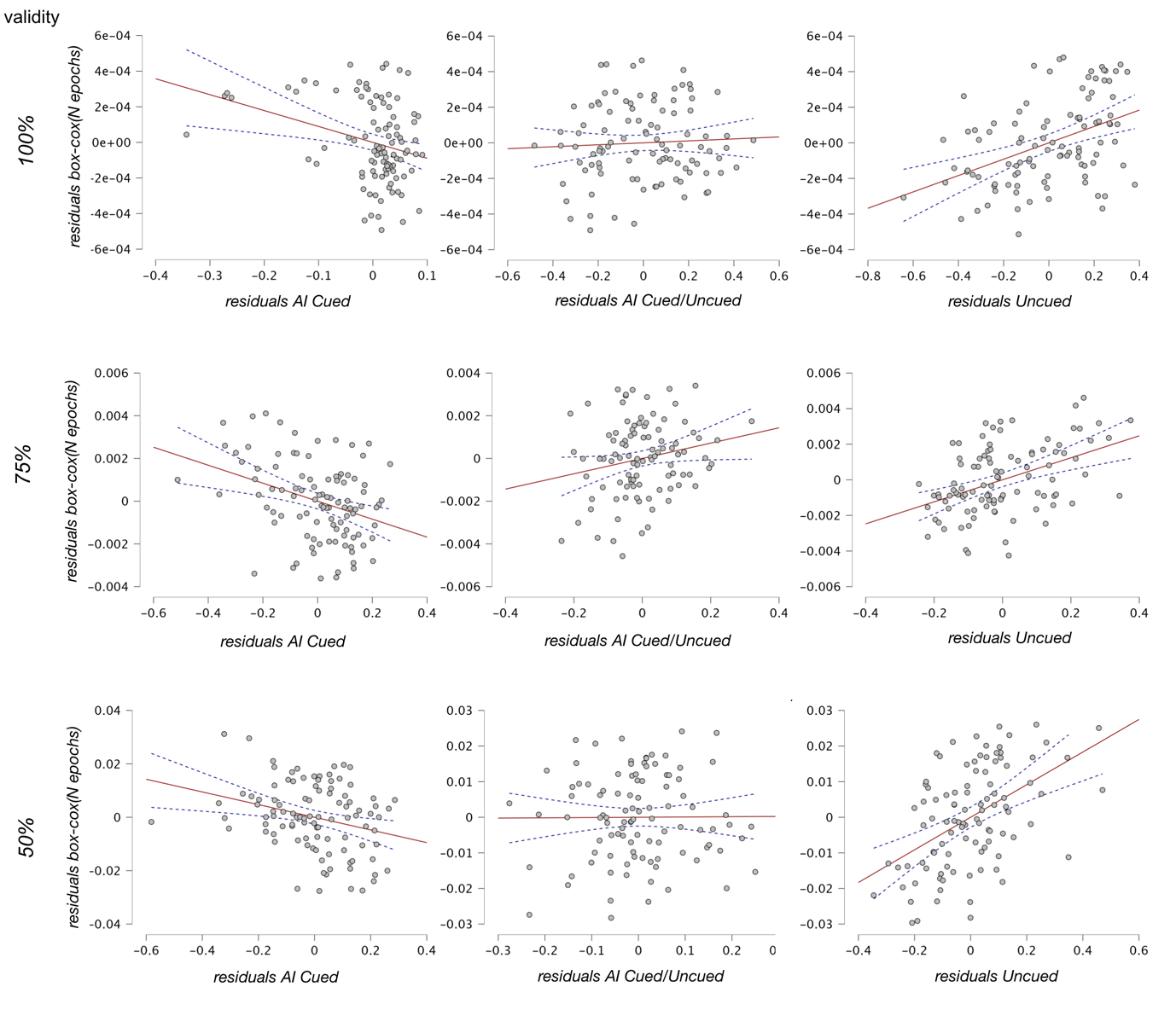


**S4 Fig.** Results of the regression analyses predicting the number of training epochs (after a box-cox transformation) from AI *Cued*, AI *Cued/Uncued* and AI *Uncued* (columns, left to right*)* in Experiment 4. Rows correspond to the three retrocue validity conditions examined (100, 75 and 50%, top to bottom). Data shown in grey dots, regression lines of best fit in red, 95% confidence interval for the slope in navy dashed lines.
